# Selection of the First ^99m^Tc-Labelled Somatostatin Receptor Subtype 2 Antagonist for Clinical Translation—Preclinical Assessment of Two Optimized Candidates

**DOI:** 10.3390/ph14010019

**Published:** 2020-12-28

**Authors:** Melpomeni Fani, Viktoria Weingaertner, Petra Kolenc Peitl, Rosalba Mansi, Raghuvir H. Gaonkar, Piotr Garnuszek, Renata Mikolajczak, Doroteja Novak, Urban Simoncic, Alicja Hubalewska-Dydejczyk, Christine Rangger, Piriya Kaeopookum, Clemens Decristoforo

**Affiliations:** 1 Division of Radiopharmaceutical Chemistry, University Hospital Basel, Universitätsspital Basel, CH-4031 Basel, Switzerland; Melpomeni.Fani@usb.ch (M.F.); rosalba.mansi@usb.ch (R.M.); raghuvirharidas.gaonkar@usb.ch (R.H.G.); 2 Department of Nuclear Medicine, Medical University Innsbruck, 6020 Innsbruck, Austria; viktoria.weingaertner@gmx.de (V.W.); Christine.rangger@i-med.ac.at (C.R.); gamsuk@hotmail.com (P.K.); 3 Department of Nuclear Medicine, University Medical Centre Ljubljana, University of Ljubljana, 1000 Ljubljana, Slovenia; petra.peitl@kclj.si (P.K.P.); doroteja.novak@kclj.si (D.N.); 4 Radioisotope Centre POLATOM, National Centre for Nuclear Research, 05-400 Otwock, Poland; Piotr.Garnuszek@polatom.pl (P.G.); Renata.Mikolajczak@polatom.pl (R.M.); 5 Faculty of Mathematics and Physics, University of Ljubljana, 1000 Ljubljana, Slovenia; urban.simoncic@ijs.si; 6 Department of Endocrinology, Jagiellonian University Medical College, 31-008 Cracow, Poland; alicja.hubalewska@gmail.com

**Keywords:** somatostatin receptor antagonist, SST2, Tc-99m, SPECT/CT, neuroendocrine tumours

## Abstract

Recently, radiolabelled antagonists targeting somatostatin receptors subtype 2 (SST2) in neuroendocrine neoplasms demonstrated certain superior properties over agonists. Within the ERA-PerMED project “TECANT” two ^99m^Tc-Tetramine (N4)-derivatized SST2 antagonists (TECANT-1 and TECANT-2) were studied for the selection of the best candidate for clinical translation. Receptor-affinity, internalization and dissociation studies were performed in human embryonic kidney-293 (HEK293) cells transfected with the human SST2 (HEK-SST2). Log *D*, protein binding and stability in human serum were assessed. Biodistribution and SPECT/CT studies were carried out in nude mice bearing HEK-SST2 xenografts, together with dosimetric estimations from mouse-to-man. [^99m^Tc]Tc-TECANT-1 showed higher hydrophilicity and lower protein binding than [^99m^Tc]-TECANT-2, while stability was comparable. Both radiotracers revealed similar binding affinity, while [^99m^Tc]Tc-TECANT-1 had higher cellular uptake (>50%, at 2 h/37 °C) and lower dissociation rate (<30%, at 2 h/37 °C). In vivo, [^99m^Tc]Tc-TECANT-1 showed lower blood values, kidney and muscles uptake, whereas tumour uptake was comparable to [^99m^Tc]Tc-TECANT-2. SPECT/CT imaging confirmed the biodistribution results, providing the best tumour-to-background image contrast for [^99m^Tc]Tc-TECANT-1 at 4 h post-injection (p.i.). The estimated radiation dose amounted to approximately 6 µSv/MBq for both radiotracers. This preclinical study provided the basis of selection of [^99m^Tc]Tc-TECANT-1 for clinical translation of the first ^99m^Tc-based SST2 antagonist.

## 1. Introduction

Combination of molecular and anatomical imaging (hybrid imaging, SPECT/CT and PET/CT) is currently the most sensitive approach for visualization of somatostatin receptor (SST)-positive tumours, in particular neuroendocrine neoplasms (NEN), utilizing radiolabelled somatostatin analogues [1,2,3]. These analogues were constructed focusing on their agonistic behaviour, based on their internalization after SST activation and consequent retention within the tumour cell, believed to be crucial for efficient molecular imaging and therapy. Over the last few years, it has been shown that novel molecular probes, SST antagonists, recognize more binding sites and hence improve the diagnostic efficacy, especially when the density of SST is low [4,5,6,7].

Preclinical data and subsequent clinical evaluation demonstrated higher tumour uptake of an indium-111 (^111^In) labelled antagonist [^111^In]In-DOTA-SST2-ANT ([^111^In]In-DOTA-BASS) compared to the agonist [^111^In]In-DTPA^0^-octreotide or [^111^In]In-DTPA^0^-octreotate, as well as superior tumour-to-background ratios [5,8]. One of the first reports describing the SST2 antagonist LM3 indicates the high potential of gallium-68 (^68^Ga) and copper-64 (^64^Cu) radiolabelled LM3 (p-Cl-Phe-cyclo(d-Cys-Tyr-d-4-amino-Phe(carbamoyl)-Lys-Thr-Cys)-d-Tyr-NH_2_) in PET/CT [6]. The authors have demonstrated strong dependence of the affinity and pharmacokinetics of the somatostatin-based radiolabelled antagonists on the chelator and radiometal, also confirmed using another SST2 antagonist, namely JR11 [9,10]. Superiority of an SST antagonist versus agonist was demonstrated in a phase I/II clinical study using the PET tracer [^68^Ga]Ga-NODAGA-JR11 [7,11] and in a pilot study conducted with the beta-emitting, therapeutic radionuclide (lutetium-177, ^177^Lu) labelled SST2 antagonist [^177^Lu]Lu-DOTA-JR11 [12]. As a result, research in the field is currently strongly focused on radiolabelled SST2 antagonists.

^68^Ga-labelled somatostatin analogues are established for PET imaging as a unique tool for personalizing treatment of NEN. Nevertheless, single-photon emitting radiopharmaceuticals still represent the cornerstone of molecular imaging, particularly those based on technetium-99m (^99m^Tc). Its physical properties (half-life of 6 h, optimal energy of 140 keV for imaging and lowest radiation exposure), widest on-site availability and cost-effectiveness are of major importance for routine clinical applications. Medical diagnostic imaging techniques using ^99m^Tc account for approximately 80% of all nuclear medicine procedures. In a recent study [13], the SST2 antagonist SS01 (p-Cl-Phe-cyclo(d-Cys-Tyr-d-Trp-Lys-Thr-Cys)-d-Tyr-NH_2_), based on the first radiolabelled SST2 antagonist applied in humans (BASS, bearing a p-NO_2_-Phe in position 1 instead of p-Cl-Phe), was conjugated to two different chelating systems, both suitable for radiolabelling with ^99m^Tc. In contrast to the hydrazinonicotinamide/ethylenediaminediacetic acid (HYNIC/EDDA) conjugate that lost its affinity for SST2, the tetramine-chelator N4 conjugate revealed high cellular uptake in SST2-expressing cells, and was clearly superior, again confirming the importance of the chelator’s choice in the development of radiolabelled SST2 antagonists. ^99m^Tc-N4-SS01 was evaluated in vivo in nude mice bearing SST2-expressing xenografts, showing an impressive tumour uptake of 47% IA/g at 4 h post-injection (p.i.) and high tumour to normal organ ratios.

**Scheme 1:**
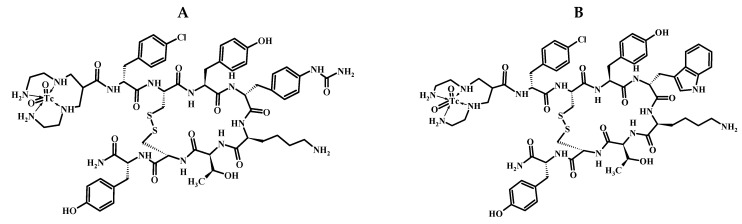
The chemical structure of [^99m^Tc]Tc-TECANT-1 (**A**) and [^99m^Tc]Tc-TECANT-2 (**B**).

The main aim of this work was to study head-to-head the two ^99m^Tc-labelled SST2 antagonists (see Scheme 1), one based on the LM3 structure (“TECANT-1” [6]), the other based on SS01 structure (“TECANT-2” [13]), both conjugated with the N4-chelator, providing a positively charged dioxo-^99m^Tc(V)-complex. These two candidates differ only on the amino acid in position four of the peptide sequence; d-4-amino-Phe(carbamoyl) in TECANT-1 vs. Trp in TECANT-2. The goal was to select the best ^99m^Tc-labelled SST2 antagonist for clinical translation in NEN imaging.

## 2. Results

### 2.1. Radiolabelling and Stability

**Figure 1:**
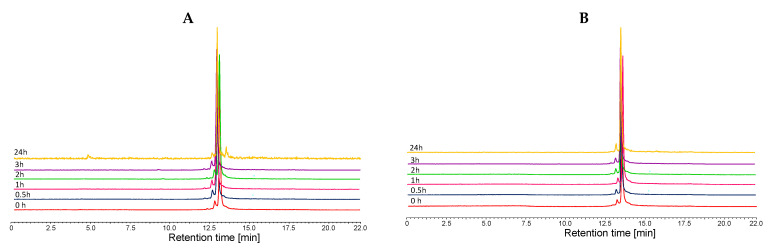
Stability of [^99m^Tc]Tc-TECANT-1 (**A**) and [^99m^Tc]Tc-TECANT-2 (**B**) in human serum at 37 °C after an incubation of 0, 30, 60, 120, 240 min, and 24 h.

TECANT-1 and TECANT-2 were labelled quantitatively with ^99m^Tc (<1% free ^99m^Tc) with an apparent molar radioactivity ranging from 20–45 MBq/nmol. Both [^99m^Tc]Tc-TECANT-1 and [^99m^Tc]Tc-TECANT-2 showed very high stability over a period of 24 h in human serum at 37 °C (Figure 1).

### 2.2. Lipophilicity and Protein Binding

The results of the lipophilicity and protein binding are presented in Table 1. [^99m^Tc]Tc-TECANT-1 bearing the d-Aph(Cbm) residue at position eight (numbering on the SS-14 sequence) showed significantly increased hydrophilicity compared to [^99m^Tc]Tc-TECANT-2 bearing d-Trp (log *D* = −2.53 ± 0.19 vs. −1.63 ± 0.16, respectively). Both radiolabelled peptides showed a constant protein binding over 4 h (approximately 20%), with [^99m^Tc]Tc-TECANT-2 having a trend towards higher binding, compared to [^99m^Tc]Tc-TECANT-1, at all investigated time points, without resulting in significant differences.

### 2.3. In Vitro Internalization and Dissociation

**Figure 2:**
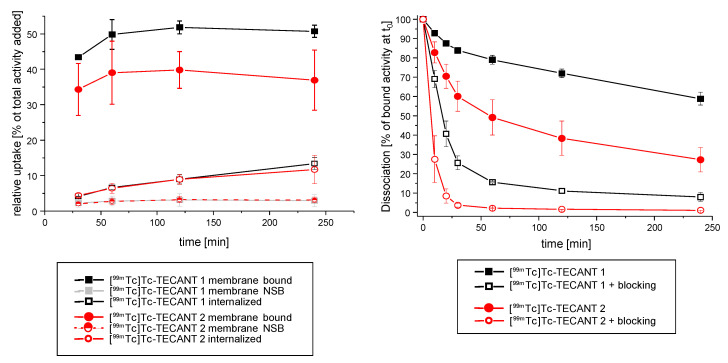
(**Left**): Cell-surface (membrane) specific bound, non-specific binding (membrane NSB) and internalization of [^99m^Tc]Tc-TECANT-1 and [^99m^Tc]Tc-TECANT-2 over time (data are shown as means ± SD for 3 independent experiments). (**Right**): Dissociation-assay of [^99m^Tc]Tc-TECANT-1 and [^99m^Tc]Tc-TECANT-2 (data are shown as means ± SD from 3 independent experiments).

The results of internalization and dissociation assays are presented in Figure 2. Both radiolabelled peptides showed high and specific (SST2-mediated) cellular binding. The main fraction was determined on the cell surface and was only slightly increased from 30 min to 4 h. [^99m^Tc]Tc-TECANT-1 showed significantly higher cell surface binding than [^99m^Tc]Tc-TECANT-2 (50.7 ± 1.7% vs. 36.9 ± 8.5%, *p <* 0.001 at 4 h, respectively). The internalized fraction was low, as expected for an antagonist, and comparable for both peptides. The internalization increased from 4.1 ± 0.1% at 30 min to 13.4 ± 1.7% at 4 h for [^99m^Tc]Tc-TECANT-1 and similarly from 4.4 ± 0.5% to 11.7 ± 1.4% for [^99m^Tc]Tc-TECANT-2. Cell-surface binding and internalization were highly specific and could be blocked with excess of ligand to approximately 3% cell membrane bound and <2% internalized fraction.

The dissociation studies showed that [^99m^Tc]Tc-TECANT-2 dissociates considerably faster than [^99m^Tc]Tc-TECANT-1 (Figure 2). Whereas total binding was still at 58.8 ± 3.3% after 4 h for [^99m^Tc]Tc-TECANT-1, it was only 27.2 ± 6.3% for [^99m^Tc]Tc-TECANT-2. In the presence of excess of the competitor, the bound fraction still remaining in the cells was reduced to 7.9 ± 2.2% for [^99m^Tc]Tc-TECANT-1 and to 1.1 ± 0.1% for [^99m^Tc]Tc-TECANT-2. The estimated half-lives were 98 min and 19 min for [^99m^Tc]Tc-TECANT-1 (absence and presence of competitor, respectively) and 40.3 min and 7.5 min for [^99m^Tc]Tc-TECANT-2, respectively.

### 2.4. Binding Affinity Studies

**Figure 3:**
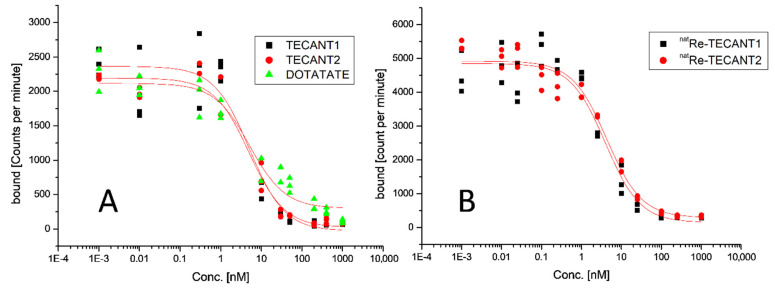
(**A**) Competition assay for determination of apparent half maximal inhibitory concentration (IC_50_) of TECANT-1 and TECANT-2, compared to DOTA^0^-Tyr^3^-Octreotate (DOTA-TATE). (**B**) Respective assay for the natural rhenium (^nat^Re) complexes.

The results of competition assays are presented in Figure 3. The mean indicative half maximal inhibitory concentration (IC_50_) values did not differ significantly and were for TECANT-1: 4.14 ± 0.83 nM, for TECANT-2: 5.00 ± 1.14 nM, comparable to DOTA^0^-Tyr^3^-Octreotate (DOTA-TATE) with 5.18 ± 1.53 nM, in the same assay. The corresponding Re-complexes revealed no alteration of the binding affinity, as compared to the non-metallated peptides, with IC_50_ values of 5.01 ± 0.93 nM and 4.47 ± 0.89 nM for ^nat^Re-TECANT-1 and ^nat^Re-TECANT-2, respectively.

### 2.5. Biodistribution

The biodistribution results of [^99m^Tc]Tc-TECANT-1 and [^99m^Tc]Tc-TECANT-2 are summarized in Table 2. [^99m^Tc]Tc-TECANT-1 showed high accumulation in SST2-expressing tumours at 1 h p.i. with the highest uptake observed at 4 h p.i. (21.03 ± 3.92 and 26.01 ± 1.33% IA/g (*p* = 0.0795), respectively). As expected for a high SST2-affinity analogue, the uptake was also high in the SST-positive organs, such as the pancreas and the stomach. The washout from these tissues though is much quicker compared to the tumour, leading to improved tumour-to-non-tumour organ ratios over time. The specificity of [^99m^Tc]Tc-TECANT-1 in vivo was proven by the negligible uptake of the radiotracer in SST2-negative tumours vs. the SST2-positive tumours at 4 h p.i. (0.88 ± 0.37 and 26.01 ± 1.33% IA/g, *p* < 0.0001). No significant difference was found in the tumour uptake between [^99m^Tc]Tc-TECANT-1 and [^99m^Tc]Tc-TECANT-2, at 1 and 4 h p.i. [^99m^Tc]Tc-TECANT-2 had a maximum uptake in the SST2-expressing tumours at 4 h p.i., similarly to [^99m^Tc]Tc-TECANT-1 (28.41 ± 4.84 vs. 26.01 ± 1.33% IA/g, *p* = 0.4015, respectively). However, at 24 h p.i., [^99m^Tc]Tc-TECANT-2 had significantly lower retention in the tumour than [^99m^Tc]Tc-TECANT-1 (10.98 ± 4.69 vs. 19.02 ± 2.00% IA/g (*p* = 0.0823), respectively). In addition, the uptake of [^99m^Tc]Tc-TECANT-2 in the pancreas and the stomach, being SST-expressing organs, was lower than [^99m^Tc]Tc-TECANT-1, while at the same time its uptake in the lung and liver was significantly higher, at 1 and 4 h p.i. Another striking difference was observed in the kidney uptake. [^99m^Tc]Tc-TECANT-2 had 2.7-times higher kidney uptake than [^99m^Tc]Tc-TECANT-1 at 1 h p.i. (43.63 ± 11.37 vs. 16.31 ± 1.83% IA/g, *p* = 0.002, respectively), which remained higher (1.4-times) 24 h later. Additionally, for [^99m^Tc]Tc-TECANT-2, a high specificity in vivo was proven by the very low uptake in SST2-negative tumours vs. the SST2-positive tumours at 4 h p.i. (0.38 ± 0.13 and 28.41 ± 4.84% IA/g, *p* < 0.0001).

### 2.6. SPECT/CT Images

**Figure 4:**
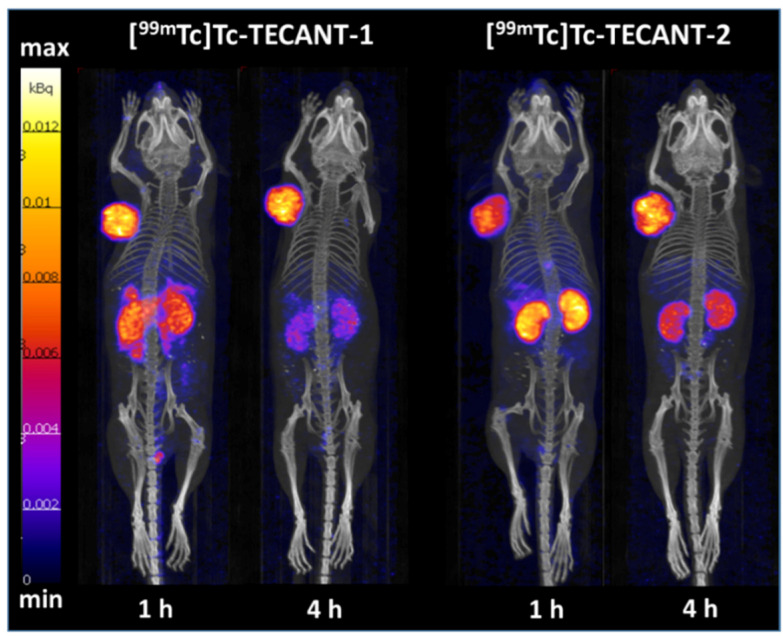
Head-to-head SPECT/CT images of [^99m^Tc]Tc-TECANT-1 and [^99m^Tc]Tc-TECANT-2 (200 pmol/5–6 MBq) in nude mice bearing HEK-SST2 (+) xenografts at 1 and 4 h p.i.

The SPECT/CT images at 1 h and 4 h after intravenous administration of [^99m^Tc]Tc-TECANT-1 and [^99m^Tc]Tc-TECANT-2 are presented in Figure 4, and they are reflecting the biodistribution data. Tumour-to-background image contrast is very high for both radiotracers. Tumour uptake is visually similar for [^99m^Tc]Tc-TECANT-1 and [^99m^Tc]Tc-TECANT-2 at 1 and at 4 h p.i. Comparatively, significantly higher kidney uptake of [^99m^Tc]Tc-TECANT-2 and more abdominal uptake for [^99m^Tc]Tc-TECANT-1 at 1 h p.i. is also seen. Four hours p.i., abdominal uptake is not visible with [^99m^Tc]Tc-TECANT-1, while the higher kidney uptake of [^99m^Tc]Tc-TECANT-2 is still obvious. Between the two radiotracers, the highest tumour-to-background contrast, including tumour-to-kidney, was achieved with [^99m^Tc]Tc-TECANT-1 at 4 h p.i.

### 2.7. Dosimetry

To account for various possible locations of the tumour, we used two S-values. One was the value for the liver and is representative for the liver lesions. Other was the “average abdominal S-value”, which was the average S-value for the following organs: stomach, liver, gallbladder, spleen, pancreas, small intestine, kidneys, large intestine, and adrenal glands. Estimated dose to various organs, average total body dose and effective dose are summarized in Table 3 for both radiotracers and two typical tumour locations.

## 3. Discussion

Somatostatin analogues, labelled with γ-emitters (e.g.,^111^In, ^99m^Tc) or β^+^-emitters (e.g., ^68^Ga, ^64^Cu), are nowadays widely used for the detection of the primary tumour, staging, restaging and assessment of treatment response in patients with NENs [2]. Despite the recent and increasing use of PET-radiopharmaceuticals due to the higher sensitivity and resolution of the PET images as compared to SPECT, ^99m^Tc remains a backbone for diagnostic procedures in nuclear medicine. The combination of its optimal nuclear properties and the diverse coordination chemistry render it highly versatile, able to be incorporated in different chelate systems at different oxidation states, and thus modulating the biological and chemical properties of the radiopharmaceutical. Even though the ^99m^Tc-based somatostatin agonist hydrazinonicotinamide/ethylenediamine *N*,*N*′-diacetic acid-Tyr^3^-octreotide (HYNIC/EDDA-TOC) is used in several European countries (^99m^Tc-Tektrotyd^®^), few preclinical studies have reported the development of somatostatin receptor antagonists radiolabelled with ^99m^Tc [13,14,15,16].

In this work, the two SST2 antagonists [^99m^Tc]Tc-TECANT-1 and [^99m^Tc]Tc-TECANT-2 were assessed comparatively to determine the best candidate for potential clinical translation within the international ERA-PerMED project “TECANT” [17]. ^99m^Tc-labelling was simple for both peptides and resulted in reproducible high yields. Details on radiolabelling results, analytics related to all potential impurities and the development of a kit-formulation will be described in a separate paper. No remarkable differences were found in the stability and the competition assays. Each radiotracer remained intact in serum at 37 °C over a period of 24 h. The affinity for SST2 was comparable for both (IC_50_ = 4.1 vs. 5.0 nM for TECANT-1 and TECANT-2, respectively). In addition, metal complexing did not alter SST2 affinity significantly, which could be shown by using the respective rhenium complexes as surrogates for ^99m^Tc.

The in vitro studies on HEK-SST2 intact cells depicted a similar, but not identical, behaviour of both radiotracers. [^99m^Tc]Tc-TECANT-1 showed higher cell surface binding and slower dissociation rate, indicating a stronger receptor–peptide interaction as compared to [^99m^Tc]Tc-TECANT-2. The low internalization rate is indicative of their antagonistic behaviour. For [^99m^Tc]Tc-TECANT-1, no direct comparison with literature data is possible since it is the first time that the peptide LM3 coupled to the chelator N4 has been evaluated. Interestingly, our data revealed that this peptide—known to be highly susceptible to diverse chelate systems [9]—in this conjugated form (N4-LM3) retained high affinity and binding properties on HEK-SST2 expressing cells, in vitro and in vivo. In addition, our data regarding the in vitro performance of [^99m^Tc]Tc-TECANT-2 on HEK-SST2 cells are in agreement with Abiraj et al. [13].

Pharmacokinetic studies showed clear differences between the two radiotracers. [^99m^Tc]Tc-TECANT-1 presented 2.9 and 2.5 higher uptake in the pancreas and stomach, respectively, (both being SST-positive) as compared to [^99m^Tc]Tc-TECANT-2, but 2.7 lower kidney uptake at 1 h p.i.. The highest tumour uptake was reached for both radiotracers at 4 h p.i. being very similar (26.01 ± 1.33% IA/g and 28.41 ± 4.84% IA/g for [^99m^Tc]Tc-TECANT-1 and [^99m^Tc]Tc-TECANT-2, respectively). Nevertheless, [^99m^Tc]Tc-TECANT-1 showed longer tumour retention with 19.02 ± 2.00% IA/g uptake at 24 h versus the 10.98 ± 4.69% IA/g of the [^99m^Tc]Tc-TECANT-2, even though not statistically significant. Imaging studies performed at 1 h and 4 h p.i. for both radiotracers reflected the biodistribution data: [^99m^Tc]Tc-TECANT-1 showed higher abdomen uptake at 1 h, already washed out at 4 h p.i., while [^99m^Tc]Tc-TECANT-2 presented higher and more persistent kidney uptake. The lower kidney uptake and fast washout of the radioactivity from the target tissues compared to the accumulation in the tumour led to better tumour-to-background contrast for [^99m^Tc]Tc-TECANT-1 at 4 h p.i., as shown in the SPECT/CT images.

The biodistribution profile of [^99m^Tc]Tc-TECANT-2 reflected the results published by Abiraj et al. on this radiotracer ([^99m^Tc]Tc-SS04 in [13], with similar distribution pattern in target tissues as well as in excretory organs over time. The lower hydrophilicity of this radiotracer compared to [^99m^Tc]Tc-TECANT-1 (−1.63 ± 0.16 and −2.53 ± 0.19, respectively) is reflected in higher liver and blood uptake.

Few attempts to develop ^99m^Tc-based somatostatin antagonistic radiotracers have been published until now, despite the wide availability and low cost of this radionuclide. A critical parameter is the choice of the chelator that needs to fulfil certain properties, including stable complexation while maintaining high affinity and specificity of the conjugate for the targeted receptor. The work of Abiraj et al. [13] illustrated the influence of the chelator on the affinity and properties of ^99m^Tc-based SST2 antagonists. The HYNIC monodentate ligand—which is used in combination with the somatostatin agonist TOC in patients ([^99m^Tc]Tc-HYNIC/EDDA-TOC)—was not a suitable chelating system for the SST2 antagonist SS01, as it led to loss of affinity. SS01 restored its affinity only when the chelator N4 was used for ^99m^Tc-labelling. The Hennkens group chose, alternatively, the [^99m^Tc]Tc(CO)_3_^+^ core for labelling of the somatostatin antagonists sst_2_-ANT (BASS, pNO_2_-Phe-c(D-Cys-Tyr-D-Trp-Lys-Thr-Cys)-D-Tyr-NH_2_) based on bifunctional chelators such as [N,S,N] [14], or [N,S,O] [15] and the polycarboxylate-derivatives of 1,4,7-triazacyclononane (TACN), such as NODAGA and NOTA [16]. Among all [^99m^Tc]Tc(CO)_3_-labelled sst2-ANT complexes, the [^99m^Tc]Tc-NOTA-sst2-ANT and [^99m^Tc]Tc-NODAGA-sst2-ANT showed better biodistribution patterns, compared to the other complexes, presumably due to their higher hydrophilicity. More precisely, [^99m^Tc]Tc-NOTA-sst2-ANT demonstrated the highest tumour uptake (16.70 ± 3.32% IA/g at 1 h p.i.) in AR4-2J tumour bearing mice, but also higher accumulation in the liver and the abdomen, compared to [^99m^Tc]Tc-NODAGA-sst2-ANT. On the other hand, the most hydrophilic [^99m^Tc]Tc-NODAGA-sst2-ANT showed significantly lower tumour uptake (2.78 ± 0.27% IA/g vs. 16.70 ± 3.32% IA/g at 1 h p.i., respectively), leading to reduced tumour-to-background ratios (i.e., tumour-to-blood and tumour-to-kidneys) [18]. The chelator of choice in this work, namely the 6-carboxy-1,4,8,11-tetraazaundecane (N4), forms a [O=Tc=O]^+^ core that exhibits high kinetic stability and hydrophilicity, while allowing easy and convenient labelling at room temperature. These features, taken together with the high tumour uptake and favourable in vivo distribution, render [^99m^Tc]Tc-TECANT-1 and [^99m^Tc]Tc-TECANT-2 suitable candidates for clinical translation.

The dosimetry study predicts low radiation dose to the human for [^99m^Tc]Tc-TECANT-1 and [^99m^Tc]Tc-TECANT-2, independent of the exact location of the tumour in the abdomen. The estimated radiation dose amounted to approximately 6 µSv/MBq for both radiotracers. For typical injected activity of 740 MBq, the effective dose is 4.9 mSv and 4.3 mSv for [^99m^Tc]Tc-TECANT-1 and [^99m^Tc]Tc-TECANT-2, respectively. The dose to some organs is several times higher, especially the dose to kidneys, stomach, liver, and pancreas, which is not surprising, based on the biodistribution results. The dose to particular organs is also substantially different for [^99m^Tc]Tc-TECANT-1 and [^99m^Tc]Tc-TECANT-2, reflecting the biodistribution results. The resulting effective dose for both radiotracers only differs to a minor extent (12% difference), while the average total body dose is almost the same for both radiotracers (within 3%). The total body dose is two times lower than the effective dose because lower dose to the extremities (not listed in the table) affects the average dose considerably, while having little effect to the effective dose. The estimated dose to the bladder is slightly higher than the effective dose and almost the same for both radiotracers—it obviously depends considerably on the assumed bladder voiding interval and will be much higher when a longer bladder voiding interval is considered.

Overall, both radiotracers have very high tumour-to-background contrast that highlight their potential as imaging agents for SST2-expessing tumours, with some favourable properties for [^99m^Tc]Tc-TECANT-1, making it the first choice for clinical translation.

## 4. Materials and Methods

### 4.1. Radiolabelling with ^99m^Tc

TECANT-1 (N4-p-Cl-Phe-cyclo(d-Cys-Tyr-d-Aph(Cbm)-Lys-Thr-Cys)-d-Tyr-NH_2_, where d-Aph(Cbm): d-4-amino-carbamoyl-phenylalanine) and TECANT-2 (N4-p-Cl-Phe-cyclo(d-Cys-Tyr-d-Trp-Lys-Thr-Cys)-d-Tyr-NH_2_) were custom made by piChem (Raaba-Grambach, Austria). For ^99m^Tc-labelling, a solution of 0.2–0.4 mL Na[^99m^Tc]TcO_4_ (300–700 MBq, obtained from ULTRATECHNEKOW^®^ generator, Curium, Petten, The Netherlands) was added to a mixture of 25 µL phosphate buffer 0.5 M and 5 µL trisodium citrate 0.1 M, followed by the addition of TECANT-1 or TECANT-2 (20 µg, 1 mg/mL in H_2_O) and SnCl_2_ in EtOH (5 µL, 2 mg/mL). The reaction mixture (pH 11–12) was incubated at room temperature for 30 min, subsequently neutralized with 15 µL NaH_2_PO_4_ 1 M and used without further purification. Quality control was performed by radio-revesed phase (RP)HPLC (column: Jupiter 4 µm Proteo 90 Å LC; column 250 × 4.6 mm; eluents: A = H_2_O/0.1% Trifluoroacetic acid (TFA), B = Acetonitrile (ACN)/0.1%TFA; flow rate: 1 mL/min; gradient: 0–1 min 0% B; 1–15 min 0–50% B; 15–16 min 80% B; 16–22 min 0% B).

### 4.2. Lipophilicity

Determination of the distribution coefficient (log *D*) was performed by the shake-flask method in 1:1 mixture of octanol/Phosphate buffered saline (PBS). Then, 50 µL of [^99m^Tc]Tc-TECANT-1 or [^99m^Tc]Tc-TECANT-2 solution was diluted up to 500 µL with PBS (pH 7.4; 8 mM phosphate). Next, 20 µL of this solution was added to six eppendorf tubes, each containing 500 µL octanol and 480 µL PBS. The samples were vortexed for 15 min at 1400 rpm followed by centrifugation for 2 min at 4500 rpm. Aliquots of 100 µL from each phase were taken and measured in a γ-counter. The log *D* was calculated as the average of the logarithmic values (*n* = 3, six technical replicates) of the ratio between the radioactivity in the octanol and the PBS phase.

### 4.3. Protein Binding

Protein binding was determined for each radiotracer by size exclusion chromatography in triplicates. A solution of [^99m^Tc]Tc-TECANT-1 or [^99m^Tc]Tc-TECANT-2 (50 µL, 10 µM) was added to 450 µL freshly prepared human serum and to 450 µL PBS (control). The mixtures were incubated at 37 °C for 30, 60, 120 and 240 min. Aliquots of 25 µL were transferred to illustra MicroSpin G-50 columns (GE Healthcare Vienna, Austria) and centrifuged for 2 min at 2000 relative centrifugal force (rcf). The eluate (protein-bound) and the column fractions (non-protein bound) were measured in a γ-counter.

### 4.4. Stability in Human Serum

The stability of [^99m^Tc]Tc-TECANT-1 and [^99m^Tc]Tc-TECANT-2 was tested in human serum, mixed as described above, at 37 °C for 0, 30, 60, 120, 240 min, and 24 h. At each preselected time-point, equal parts of the incubation solution and methanol were mixed in eppendorf tubes and centrifuged for 2 min at 14,000 rpm. Fifty µL of the supernatant was transferred in a separate eppendorf tube, containing the same amount of water, and analysed by radio-RP-HPLC (2 replicates).

### 4.5. Cell Culture

The human embryonic kidney-293 (HEK293) cell line expressing the T7-epitope tagged human SST_2_ receptor (HEK-SST2) was provided by Professor Stefan Schulz (Institute of Pharmacology and Toxicology, Jena University Hospital, Jena, Germany). The cells were cultured at 37 °C and 5% CO_2_ in Dulbecco’s modified Eagle’s medium (DMEM) supplement with 10% fetal bovine serum (FBS), 1% penicillin-streptomycin (10,000 units—10 mg/mL), 2% l-Glutamin (200 mM) and 1% G418 (50 mg/mL). For all cell experiments, HEK-SST2 were seeded at a density of 10^6^ cells/well in 6-well plates and incubated over night with culture medium, containing 1% fetal bovine serum (FBS) to obtain a good cell adherence. The plates were pre-treated with a solution of 10% poly-lysine to promote cell adherence. Non-transfected HEK cells (SST2-negative) were used as negative control.

### 4.6. Internalization

On the day of the experiment, the medium was removed from the plates, the cells were washed with PBS and were incubated with fresh medium (DMEM with 1% FBS) for 1 h at 37 °C/5% CO_2_. [^99m^Tc]Tc-TECANT-1 or [^99m^Tc]Tc-TECANT-2 (final concentration 0.25 nM) was added to the medium and the cells were incubated (in triplicates) for 0.5, 1, 2 and 4 h at 37 °C/5% CO_2_. The internalization process was stopped by removing the medium and washing the cells twice with ice-cold PBS. The cells were then treated twice for 5 min with ice-cold glycine solution (0.05 M, pH 2.8), to distinguish between cell surface-bound (acid releasable) and internalized (acid resistant) radiotracer. Finally, the cells were detached with NaOH 1 M at 37 °C and washed twice with NaOH 1 M. To determine non-specific cellular uptake, selected wells were incubated with the [^99m^Tc]Tc-TECANT-1 or [^99m^Tc]Tc-TECANT-2 in the presence of 0.25 µM TECANT-1 or TECANT-2, respectively. The results are expressed as percentage of the total radioactivity added (% of surface bound radioactivity for the glycine fraction and % of internalized for the NaOH fraction).

### 4.7. Dissociation of the Cell-Surface Bound [^99m^Tc]Tc-TECANT-1 and [^99m^Tc]Tc-TECANT-2 with or without Competitors

The dissociation of [^99m^Tc]Tc-TECANT-1 and [^99m^Tc]Tc-TECANT-2 was studied by replacing the media at each time point, with or without the addition of high excess of the competitors TECANT-1 and TECANT-2, respectively. HEK-SST2 cells were seeded on 6-well plates (10^6^ cells/well) that were placed on ice for 30 min, before starting the experiment. The radiotracers were added to the medium (final concentration 0.25 nM) and allowed to bind to the cells at 4 °C, in order to prevent internalization. After incubation for 2 h, the cells were quickly washed twice with ice-cold PBS to remove the unbound radiotracer, followed by the addition of 1 mL pre-warm medium (with or without the competitor) and incubated at 37 °C for 10, 20, 30, 60, 120, and 240 min. At the pre-selected time points, the medium was removed for quantification and it was replaced by fresh pre-warmed (37 °C) medium (with or without 2.5 µM concentration of the respective TECANT analogue, as competitor). At the end of the experiment, the cells were solubilized with NaOH 1 M, and collected for quantification of the remaining cell-associated radiotracer (cell-surface bound and internalized fractions).

### 4.8. Rhenium-TECANT-1 and -2 Complexes

The Re-complexes of TECANT-1 and TECANT-2 were synthesized as surrogates of the Tc-counterparts. TECANT-1 or TECANT-2 (100 µL, 1 mg/mL in ethanol), tetrabutylammonium-tetrachloro-oxo-rhenate (V) (25 µL, 5 mg/mL in ethanol), 10 µL diisopropylethylamine and 10 µL trisodium citrate solution (0.5 M in water) were incubated for 30 min at room temperature. Subsequently, the reaction was stopped by neutralizing the pH with 10 µL acetic acid. The Re-complexes were analysed by RP-HPLC (see Appendix A Figure A2 and Figure A3) and by electrospray ionization mass spectrometry (ESI-MS).

### 4.9. Comparative Apparent Binding Affinity Studies (IC_50_)

The receptor binding assay was performed on 96-well MultiScreen Filter Plates HTS (1 μm glass fibre filter, Merck Millipore, Darmstadt, Germany), using [^177^Lu]Lu-DOTA-TATE as radioligand and TECANT-1, TECANT-2, ^nat^Re-TECANT-1, ^nat^Re-TECANT-2 or DOTA-TATE as competitors. After moistening the plate with TRIS buffer, 5 × 10^5^ HEK-SST_2_ cells (100 µL), 50 µL of the competitor in 8–10 different concentrations (ranging from 0.004 to 4000 nM) and 50 µL of [^177^Lu]Lu-DOTA-TATE (1 nM) were added to each well. The plate was incubated for 2 h at 4 °C. The supernatant was then removed by vacuum and the plate was washed twice with ice-cold PBS/0.1% bovine serum albumin (BSA). The filters were collected, and the remaining activities were measured in a gamma counter. The apparent IC_50_ values were calculated using a nonlinear curve fitting in OriginPro 6.1 software (Northampton, MA, USA) from 3 replicates.

### 4.10. Biodistribution Studies of [^99m^Tc]Tc-TECANT-1 and [^99m^Tc]Tc-TECANT-2

Animal experiments were approved by the authorities in accordance with the Swiss regulations for animal treatment (approval no. 2799). Female athymic nude-*Foxn1^nu^/Foxn1^+^* mice (Envigo, The Netherlands), 4–6 weeks old, were inoculated subcutaneously with 10^7^ HEK-SST2 cells on the shoulder, freshly suspended in 100 μL sterile PBS. One group of mice was inoculated, in addition, with 10^7^ HEK cells (SST2-negative) on the other shoulder. The tumours were allowed to grow for approximately 3 weeks, until they reached an average weight of approximately 250–300 mg.

Quantitative biodistribution studies were conducted with [^99m^Tc]Tc-TECANT-1 and [^99m^Tc]Tc-TECANT-2 (100 µL/20 pmol/0.3–0.6 MBq) at 1, 4 and 24 h p.i. The organs of interest were collected, rinsed, blotted, weighed, and counted in a γ-counter. The results are expressed as percentage of injected activity per gram (%IA/g) obtained by extrapolation from counts of an aliquot taken from the injected solution as a standard. The specificity of the uptake of [^99m^Tc]Tc-TECANT-1 and [^99m^Tc]Tc-TECANT-2 was assessed 4 h p.i. in dual HEK-SST2/HEK (SST_2_-negative) xenografted mice.

### 4.11. SPECT/CT Imaging Study

Mice bearing HEK-SST2 tumours were euthanized 1 h and 4 h after intravenous injection of 100 µL/200 pmol/5–6 MBq) of [^99m^Tc]Tc-TECANT-1 and [^99m^Tc]Tc-TECANT-2 and imaged supine, head first, using a SPECT/CT system dedicated to imaging small animals (NanoSPECT/CT^TM^ Bioscan, Mediso Medical Imaging Systems, Budapest, Hungary). Topogram and helical CT scan of the whole mouse were first acquired using the following parameters: X-ray tube current: 177 µA, X-ray tube voltage: 45 kVp, 90 s and 180 frames per rotation, pitch 1. The helical SPECTscan was then acquired from head to toe using multi-purpose pinhole collimators (APT1). The energy window width was 20% centred symmetrically over the energy peak of ^99m^Tc at 140 keV. Twenty-four projections (200 s per projection) were used, allowing the acquisition of at least 50 kilocounts/projections.

SPECT images were reconstructed iteratively and filtered using the software package (HiSPECT v1.4.1876, SciVis GmbH, Goettingen, Geramny) and the manufacturer’s algorithm (3 subsets, 9 iterations, 35% post filtering, 128 × 128 matrix, zoom 1, 30 × 20 mm transaxial field of view, resulting in a pixel size of 0.3 mm). CT images were reconstructed using CTReco version r1.146 (Bioscan, Mediso Medical Imaging Systems, Budapest, Hungary), with a standard filtered back projection algorithm (Exact Cone Beam) and post-filtered (RamLak, 100% frequency cut-off), resulting in a pixel size of 0.2 mm. Co-registered images were visualized in the 3 orthogonal planes and as maximal intensity projection with InVivoScope v1.43 (Bioscan, Mediso Medical Imaging Systems, Budapest, Hungary)

### 4.12. Dosimetric Calculations

Dosimetric calculations were based on the biodistribution of [^99m^Tc]Tc-TECANT-1 and [^99m^Tc]Tc-TECANT-2 (Table 3). In order to account for the differences in metabolic rate among mice and humans due to significantly different body mass, time-scaling of biodistribution data was completed according to [19]:

(1) $$t_{h}=t_{m}\times {\left[\frac{m_{h}}{m_{m}}\right]}^{\frac{1}{4}}$$

where *t_m_* is the time at which a measurement was made in mice, *t_h_* is the corresponding time assumed for the human data, and *m_m_* and *m_h_* are the total body masses of the experimental mice (on average) and of the human phantom that is then used for the dosimetry, respectively. Percent of injected activity per organ in a human model was evaluated according to the animal data extrapolation model [19]:

(2) $${\left(\frac{\%IA}{\mathrm{organ}}\right)}_{\mathrm{human}}=\left[{\left(\frac{\%IA}{g}\right)}_{\mathrm{mouse}}\times M_{\mathrm{mouse}}\left(g\right)\right]\times {\left(\frac{m\left(g\right)}{M\left(\mathrm{kg}\right)}\right)}_{\mathrm{human}}$$

Here *m* is the organ mass and *M* is the total body weight of mouse or human. Average total weight of the mouse was measured, while human phantom has the total weight of 73 kg [20]. The *%IA/*g and *%IA/*organ is the percentage of the injected activity concentration (i.e., per gram of tissue) and per organ, respectively. For the tumour, we assumed that the percent of injected activity uptake into the tumour is the same for mouse and human.

Subsequently, percentage of the injected activity per organ was folded with the decay curve of ^99m^Tc and interpolated with the exponential function. The interpolation function was used to estimate initial %ID/organ and the time-integrated activity concentration (TIAC). Absorbed dose per injected activity for various organs was calculated with the TIAC factors and S-values for a standard man from the OpenDose project [21]. Effective dose was evaluated according to the ICRP 103 recommendations.

**Figure A1:**
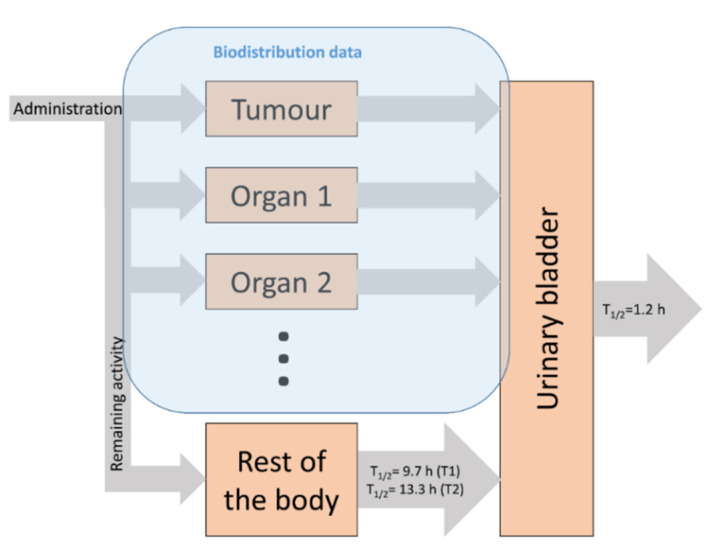
Biological pathway of the radiotracer. Biodistribution data are used to estimate the dose contribution from the organs included in the biodistribution study. Excreted radiotracers from these organs are supposed to go into the urinary bladder. The remaining activity is uniformly distributed over the rest of the body and cleared to the bladder with the biological half-life of 9.7 h for [^99m^Tc]Tc-TECANT-1 and 13.3 h for [^99m^Tc]Tc-TECANT-2. Radiotracer in the bladder has biological half-life of 1.2 h (voiding every 2.4 h).

**Figure A2:**
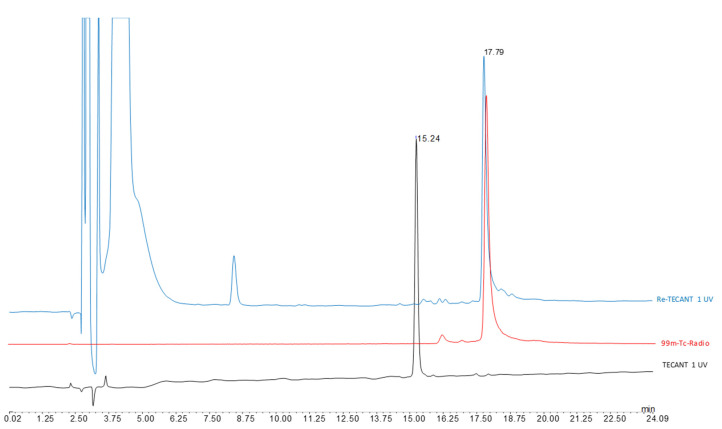
HPLC chromatogram of [^nat^Re]Re-, [^99m^Tc]Tc- and unlabelled TECANT-1 (confirmative mass spectrometry data available on request).

**Figure A3:**
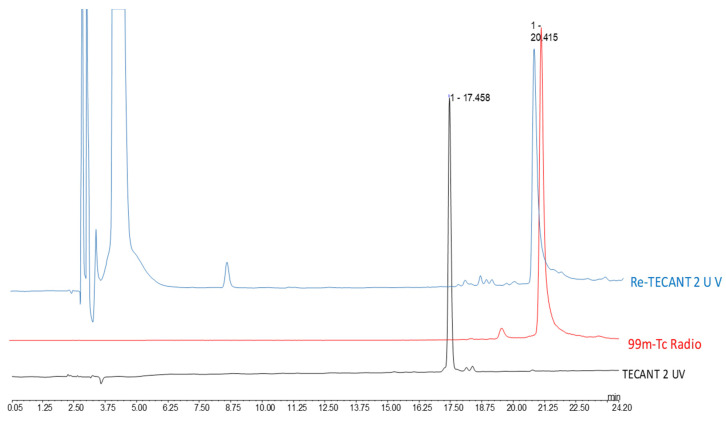
HPLC chromatogram of [^nat^Re]Re-, [^99m^Tc]Tc- and unlabelled TECANT-2 (confirmative MS data available on request).

It was assumed that all remaining activity, i.e., the activity that does not go to the organs or tumour included in the biodistribution study, is uniformly distributed over the rest of the body and cleared to the bladder with the biological half-life of 9.7 h for [^99m^Tc]Tc-TECANT-1 and 13.3 h for [^99m^Tc]Tc-TECANT-2 (estimate from the remaining activity in experimental mice, time-scaled to humans according to Equation (1)). We also assumed that the remaining activity from the organs and tumour ends up in the bladder. For the bladder, a biological half-life of 1.2 h, which roughly corresponds to voiding every 2.4 h, was assumed. Radiotracer biological pathway is presented in Figure A1 (Appendix A).

## 5. Conclusions

[^99m^Tc]Tc-TECANT-1 and [^99m^Tc]Tc-TECANT-2 showed a similar behaviour regarding their stability in human serum and their affinity for the human SST2 receptor. Nevertheless, the cellular uptake of [^99m^Tc]Tc-TECANT-1 was higher and its dissociation slower, compared with [^99m^Tc]Tc-TECANT-2. The in vivo comparison indicates better performance of [^99m^Tc]Tc-TECANT-1 due to the longer tumour residence time and the lower kidney and liver uptake, whereas estimated human radiation dose was similar for both radiotracers. Overall, this head-to-head preclinical evaluation provided the basis for selection of [^99m^Tc]-TECANT-1 for clinical translation as the first ^99m^Tc-labelled SST2 antagonist. Toxicity studies are planned to complete the preclinical characterisation of this radiotracer and the development of a kit formulation (to be reported separately) is on-going for the translation of [^99m^Tc]Tc-TECANT-1 into a clinical feasibility study in NEN patients.

## Figures and Tables

**Table 1:** Protein binding and log *D* of [^99m^Tc]Tc-TECANT-1 and [^99m^Tc]Tc-TECANT-2.

	[^99m^Tc]Tc-TECANT-1	[^99m^Tc]Tc-TECANT-2
Time (min)	% of Protein Binding	
30	17.8 ± 4.1	22.2 ± 6.1
60	18.1 ± 1.5	20.5 ± 5.7
120	19.9 ± 4.4	22.7 ± 4.2
240	19.4 ± 6.4	22.0 ± 3.7
**log *D***	−2.53 ± 0.19	−1.63 ± 0.16

**Table 2:** Biodistribution results of [^99m^Tc]Tc-TECANT-1 and [^99m^Tc]Tc-TECANT-2 in nude mice bearing human embryonic kidney-293 (HEK293) cells transfected with the human SST2 (HEK-SST2) (positive) tumours. The cohort of 4 h post-injection (p.i.) included dual HEK-SST2 and HEK (SST2-negative) tumours. The results are expressed as %IA/g ± SD (*n* = 4–9).

	[^99m^Tc]Tc-TECANT-1			[^99m^Tc]Tc-TECANT-2		
Organ	1 h	4 h	24 h	1 h	4 h	24 h
Blood	0.79 ± 0.09	0.23 ± 0.05	0.03 ± 0.00	1.80 ± 0.44	0.23 ± 0.05	0.03 ± 0.01
Heart	0.71 ± 0.10	0.24 ± 0.06	0.05 ± 0.02	1.64 ± 0.60	0.26 ± 0.05	0.05 ± 0.01
Lung	8.94 ± 3.92	3.07 ± 1.16	0.26 ± 0.04	17.00 ± 5.75	2.52 ± 0.53	0.30 ± 0.05
Liver	3.13 ± 0.43	1.90 ± 0.41	0.60 ± 0.10	5.38 ± 1.36	3.72 ± 0.92	1.07 ± 0.24
Pancreas	47.84 ± 7.86	16.91 ± 3.97	1.08 ± 0.19	16.37 ± 4.53	1.96 ± 0.58	0.12 ± 0.05
Spleen	1.30 ± 0.41	0.57 ± 0.11	0.20 ± 0.01	1.61 ± 0.46	0.55 ± 0.15	0.24 ± 0.06
Stomach	36.55 ± 7.89	13.60 ± 3.86	1.42 ± 0.27	14.60 ± 4.89	2.17 ± 0.39	0.45 ± 0.08
Intestine	6.48 ± 4.37	2.40 ± 1.90	0.31 ± 0.08	4.05 ± 1.12	0.86 ± 0.40	0.31 ± 0.17
Adrenals	7.11 ± 3.04	4.36 ± 1.88	0.85 ± 0.18	5.14 ± 1.79	1.25 ± 0.33	0.73 ± 0.20
Kidneys	16.31 ± 1.83	12.63 ± 2.54	2.42 ± 0.20	43.63 ± 11.37	25.85 ± 5.23	3.42 ± 1.44
Muscle	0.23 ± 0.05	0.12 ± 0.06	0.05 ± 0.01	0.69 ± 0.22	0.15 ± 0.04	0.06 ± 0.03
Bone	1.41 ± 0.77	0.59 ± 0.23	0.35 ± 0.07	1.67 ± 0.94	0.41 ± 0.14	0.20 ± 0.19
Pituitary	9.71 ± 2.81	6.66 ± 3.67	4.35 ± 2.87	4.74 ± 1.50	1.21 ± 0.86	2.12 ± 1.36
HEK-SST2 (+)	21.03 ± 3.92	26.01 ± 1.33	19.02 ± 2.00	19.12 ± 4.47	28.41 ± 4.84	10.98 ± 4.69
HEK-SST2 (−)		0.88 ± 0.37			0.38 ± 0.13	

**Table 3:** Dose to various organs, average total body dose (μGy/MBq) and effective dose (μSv/MBq) for both radiotracers and two possible locations of the tumour (abdomen, liver).

	[^99m^Tc]Tc-TECANT-1		[^99m^Tc]Tc-TECANT-2	
Tumour Location	Abdomen	Liver	Abdomen	Liver
Lower large intestineI wall	7.6	6.2	6.2	5.3
Small intestine wall	1.9	1.9	1.9	1.9
Stomach	20.0	17.7	11.9	10.4
Upper large intestine wall	9.1	6.4	7.1	5.4
Heart wall	5.1	5.4	5.9	6.0
Kidneys	14.8	11.9	24.6	22.7
Liver	10.7	14.6	10.5	13.1
Lungs	7.5	7.7	10.5	10.7
Muscle	2.4	2.4	2.6	2.6
Pancreas	29.4	26.7	16.1	14.4
Red marrow	3.3	3.3	3.4	3.4
Bone	3.9	3.9	4.0	4.0
Spleen	8.2	5.0	7.6	5.4
Bladder wall	8.9	8.7	8.4	8.2
Total body	3.2	3.2	3.3	3.3
Effective dose [µSv/MBq]	6.8	6.4	5.9	5.7

## Data Availability

The data presented in this study are available in this article.
